# Platelet rich fibrin and MTA in the treatment of teeth with open apices

**DOI:** 10.1186/s12903-024-03923-5

**Published:** 2024-02-13

**Authors:** Van-Khoa Pham, Tran-Lan-Khue Pham, An-Tran Pham, Hoang-Lan-Anh Le, Thi-Bich-Van Tran, Manh-Cuong Hoang, Ta-Binh Vo, Khanh-Ngoc Vy, Minh-Hong Tran, Thi-Anh-Thu Tran, Minh-Anh Bui, Anh-Dung Hoang, Ngoc-Phuc Nguyen, Thi-Tam-Duyen Nguyen, Phuc-Nguyen Nguyen, Thi-Tuong-Vi Tran, Cao-Hoai-Linh Nguyen

**Affiliations:** 1 Faculty of Odonto-Stomatology, University of Medicine and Pharmacy at Ho Chi Minh City, Ho Chi Minh City, Vietnam; 2 National Hospital of Odonto-Stomatology, Ho Chi Minh City, Vietnam; 3 Faculty of Dentistry, Van Lang University, Ho Chi Minh City, Vietnam; 4 Faculty of Odonto-Stomatology, Tra Vinh University, Tra Vinh, Vietnam; 5 Hospital of Odonto-Stomatology, Ho Chi Minh City, Vietnam

**Keywords:** PRF, MTA, Apical barrier, Open apex, Periapical periodontitis

## Abstract

**Background:**

The present study aimed to evaluate the effectiveness of using platelet-rich fibrin (PRF) as the apical matrix for the placement of MTA in nonsurgical endodontic therapy for teeth with periapical lesions and open apices.

**Methods:**

Twelve teeth from eleven patients with periapical periodontitis and open apices were enrolled in the study. Nonsurgical endodontic therapy was performed with the PRF used as an apical barrier and the MTA manipulated as an apical plug for further thermoplasticized gutta percha in the remaining part of the root canal. Clinical signs and periapical digital radiographs were recorded and analyzed to evaluate the curing progress after periodical follow-ups of 1, 3, and 6 months. The horizontal dimension of the periapical lesion was determined, and the changes in the dimensions were recorded each time. The Friedman test was used for statistical analysis, with *P* < .05 serving as the threshold for determining statistical significance.

**Results:**

All patients had no clinical symptoms after the first month of treatment, with a significant reduction in the periapical lesion after periodical appointments.

**Conclusions:**

PRF is an effective barrier when combined with MTA for the treatment of teeth with periapical periodontitis and open apices.

## Background

Teeth with open apices and periapical lesions because of traumatic or abnormal reasons have been endodontically treated by many methods, from conventional apexification with calcium hydroxide to modern application of MTA [1].

The traditional nonsurgical approach for apexification using calcium hydroxide Ca(OH)_2_ has certain drawbacks, such as prolonged follow-up, multiple visits, patient compliance requirements, difficulty, and distinctive manipulation [2]. Although this conventional method is not a sensitive technique, the weakened dentin structure could be an unexpected outcome of this uncomplicated measurement. Patient compliance is paramount for the success of this long-term process because of the possibility of reinfection of the root canal space resulting from inaccurate follow-up checks, accidentally broken temporary filling or even the remaining weak tooth structure [3].

Apical closure using MTA on the canal cervical third and a blood clot in the whole root canal space for revascularization of the pulp complex connective tissue is also a promising option [4]. However, this sensitive technique has unpredictable outcomes with certain unfavorable consequences.

Both of the above methods are not suitable for severely missing dental structure teeth, where immediate requirements for restoration are urgently needed. Occlusal situation and aesthetic issues are also important factors in determining the choice of these two treatment options. If these circumstances are unfavorable, apexification and apical closure with regeneration of the pulp complex are not proper choices for the operator.

The prevention of apical extrusion of material is a considerable challenge for teeth with wide apices and periapical lesions. In addition to being wet and dark, substances are easily removed from the apex foramen because of the open space, especially for slurries and substances with extensive setting times, such as calcium silicate-based cement and endodontic sealers. Although the apical extrusion of MTA has an unconsiderable effect on the healing process of periapical lesions, healing progress takes longer, and this outcome was not recommended by the authors of a previous study [5].

Chemical or thermal gutta-percha for root canal obturation of the apical third is an ineffective and unreliable technique for accessing teeth with open apices, especially those with severe periapical lesions [1].

The formation of an apical barrier for a certain biological substance is necessary for predictable, favorable, and effective placement of calcium-silicate material such as MTA [1]. Hemostatic collagen membranes have been successfully applied to the barrier at the apical region to prevent apical extrusion of substances in previous studies [1, 6]. However, this material has not been an autogenous substance for ensuring a completely biocompatible healing process.

By introducing platelet-rich fibrin (PRF) [7], this strong fibrin membrane enriched with platelets and growth factors has become popular in dentistry, especially in endodontic therapy [8–10]. The PRF membrane has been successfully used as an apical barrier for safe MTA or Biodentine placement in previous studies [11, 12]. Leukocyte PRF (L-PRF) [13] has been used in these case reports with promising results. However, to date, advanced PRF (A-PRF) [13] has not been manipulated as an apical barrier in the endodontic treatment of teeth with open apexes and periapical lesions [14–16].

The aim of the present study was to evaluate the effectiveness of the combination of A-PRF as an apical barrier and MTA for the treatment of teeth with periapical lesions and open apices.

## Methods

This study was approved by the Research Ethics Committee of the University of Medicine and Pharmacy at Ho Chi Minh City, Vietnam, with the approval number 236/ĐHYD-HĐĐĐ. Informed consent was obtained from all participants or their parents, and all methods were performed in accordance with the relevant guidelines and regulations. Informed consent was also obtained from all patients and/or their legal guardians for the publication of identifying information/figures in an online open-access version.

The sample size was calculated using G*Power version 3.1.9.6 (Universität Kiel, Germany) with an effect size of 1.05, an alpha of 0.05, and a power of 0.9, leading to a sample size of 12 teeth. The Wilcoxon signed-rank test (matched pairs) in the software was chosen for the reduction of the horizontal dimension of the periapical radiolucent area before and after nonsurgical endodontic therapy at the periodical appointments.

A total of eleven patients with teeth on both the maxillary and mandibular sides were recruited for the present study. The inclusion criteria were teeth with a diagnosis of symptomatic or asymptomatic apical periodontitis, periapical lesions, and open apices. The exclusion criteria were patients who had orofacial chronic pain, such as migraine, sinusitis, temporomandibular pain, or trigeminal neuralgia. Teeth with severe structures that were unrestorable, had unacceptable crown-to-root ratios, or were cracked were also not included in the present study.

The subjects were recruited at the Department of Operative Dentistry and Endodontics, Faculty of Odonto-Stomatology, University of Medicine and Pharmacy at Ho Chi Minh City, Vietnam, from November 2021 to April 2023.

All endodontic procedures were performed by the same endodontist via the same standard procedure. For the first appointment, after completing the administrative procedure, a putty silicone bite registration impression was made to capture the first long cone periapical digital radiograph using the X-Mind unit and the phosphor plate (Satelec, Acteon Group, France) with a 16-inch position device.

Local anesthesia was administered using 2% lidocaine (Lignospan standard, Septodont, France), followed by rubber dam isolation (Ash Rubber Dam, Dentsply Sirona, Switzerland). A 3D dental operating microscope (Promise Vision 3D, Seiler, St. Louis, MO, USA) was used for enhanced vision and illumination during the entire nonsurgical root canal procedure.

The endodontic access cavity was prepared using Martin and Endo-Z burs (Dentsply Sirona, Maillefer, Switzerland) under copious sterile water spray. The root canal length was subsequently measured via the synthesis of information from an electronic apex locator (ProPex PiXi, Dentsply Sirona, Maillefer, Switzerland), a periapical digital radiograph, and observation under a dental microscope. The working length was shorter than the root canal length at 2 mm. The root canal was prepared using K-files (Dentsply Sirona, Maillefer, Switzerland) with gentle, circumferential motion. A Max-i-Probe needle (Dentsply Sirona, Maillefer, Switzerland) was used to deliver 3% sodium hypochlorite (Canal Pro, Coltene Whaledent, Altstätten, Switzerland) into the root canal space for copious irrigation.

Root canal preparation was performed with the purpose of at least dentin removal and as much content cleaning as possible. Saline solution was used for final irrigation of the root canal. After being parched by sterile paper points, the root canal was filled with calcium hydroxide paste (Endo Cal, Septodont, France), which was subsequently gently condensed using a proper plugger (Dentsply Sirona, Maillefer, Switzerland), ensuring that the paste fully occupied the entire root canal, from the apical end to the cemento-enamel junction. The Cavit (GC, Tokyo, Japan) was used to fill the access cavity with an underlying sterile cotton pellet.

The next appointment was scheduled for one week after the first visit. The apical plug procedure was prepared after the completion of the root canal content removal and drying. Ten milliliters of venous blood was drawn from the subjects’ venae brachia and centrifuged at 1300 rotations per minute (RPM) for 8 min at a maximal relative centrifugal force (RCF-max) of 208 g [13] using an apparatus (PRF Duo Quattro, Nice, France). The fibrin clots containing the platelets were extracted. The PRF block was then placed under the cover of the box, over the flat plane inside the box, for 120 s to allow the formation of the PRF membrane. The PRF membrane was subsequently cut into 3 mm × 3 mm pieces, inserted, and gently condensed into the apical apex region with an appropriate plugger. Under the microscope, the pieces of the cut PRF membrane were condensed incrementally through the canal into the periapical area using the proper condenser until a firm barrier was established at the apex of the root. The procedure was performed rapidly and easily because of the widening of the apical portion of the root. The position and texture of the PRF barrier were checked for stability with a plugger under a dental operating microscope, and additional pieces of PRF could be further condensed into place if needed.

MTA Angelus (Angelus, Brazil) was mixed following the manufacturer’s instructions and inserted into the MTA carrier by digging the gun into the prepared MTA material mass. The MTA material was delivered into the apical region by triggering the piston of the carrier. The plugger was used to gently pack the material against the PRF barrier until 5 mm of MTA was placed. The placement of MTA was checked for appropriateness or any material extrusion by digital radiography.

The remaining canal space was obturated with thermoplastic gutta-percha (EQV, Meta Biomed, Korea) and AH Plus sealer (Dentsply Sirona, Maillefer, Switzerland). A composite and bonding system (GC, Tokyo, Japan) was used to restore the access cavity after ensuring obturation quality via digital radiography.

**Fig. 1:**
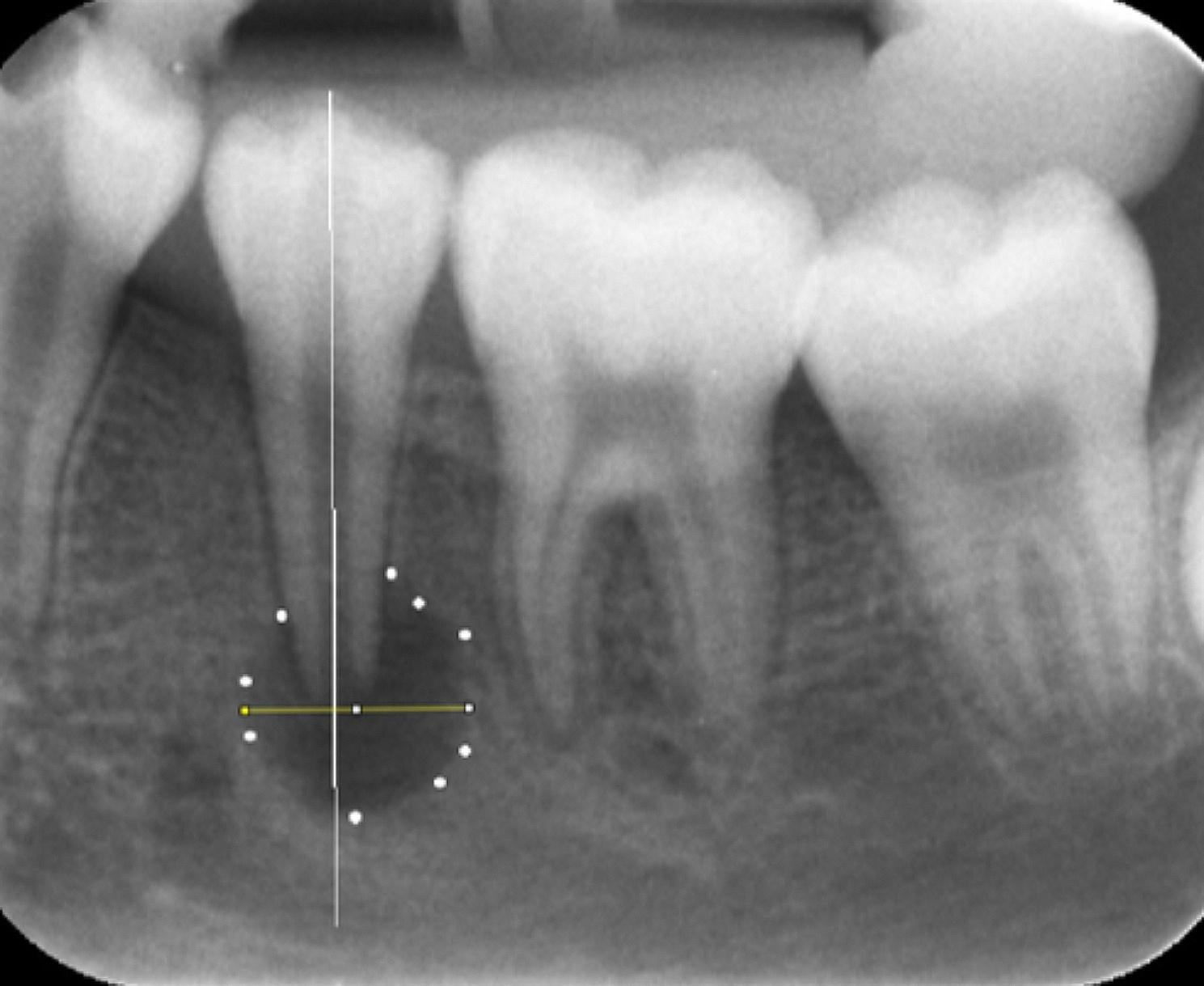
The horizontal dimension was defined as the largest distance of the segment from the two intersections between the lesion circumferential and the line drawn perpendicular to the root axis

Periodical follow-up periods of 1 month, 2 months, 3 months, and 6 months were scheduled for collecting the clinical and radiographical findings (using the individual bite registration impressions). Clinical symptoms such as postoperative pain and analgesic consumption, pain upon palpation or percussion, and sinus tract were recorded. Treatment failure was confirmed if any of the above clinical symptoms existed. Certain dimensions of the periapical lesion were measured to obtain the data for analysis. The dimension was determined as the largest distance of the segment from the two intersections between the lesion circumferential and the line drawn perpendicular to the root axis (Fig. 1). Radiographic success was confirmed if the dimension was reduced or unchanged. All measurements were conducted by the same operator, and the Kappa (for clinical symptoms) or Intra-class correlation (for radiographic dimensions) coefficient was used to evaluate intra-examiner agreement. All horizontal dimensions of the periapical lesions were remeasured by the same examiner after each two-week period, following the periodic examinations to calculate the intra-class correlation coefficients.

All the statistical analyses were performed using IBM SPSS Statistics version 27 (IBM, Armonk, NY, US), with a significance level of *p* < .05. The data were first tested for normality by the Shapiro–Wilk test or paired t test. If the data were not normally distributed, they were analyzed using the Friedman and Dunn–Bonferroni post hoc correction.

## Results

The Kappa and Intra-class correlation coefficients were greater than 0.9 for the examiner.

The horizontal dimensions of the periapical lesions on the radiographs are displayed and analyzed in Table 1.

**Table 1:** The horizontal dimensions of the periapical lesions on the digital radiographs through periodic examinations

	Minimum	25th percentile	Median	75th percentile	Maximum	Friedman test
T_0_	3.5400	4.667	5.815	7.142	9.870	< 0.00001*
T_1_	0.6700	3.277	3.455	3.907	4.890	
T_2_	0.0000	0.200	1.715	2.817	3.480	
T_3_	0.0000	0.000	0.000	0.830	2.500	
T_6_	0.0000	0.000	0.000	0.000	1.390	

T_0_, T_1_, T_2_, T_3_, and T_6_ Periapical lesion width at the beginning of treatment and at 1, 2, 3, and 6 months, respectively

* P < .05, Friedman test

There was a width of nearly ten millimeters at the diagnostic stage, and the patient was completely cured after all at 6 months. In fact, the patient healed within the first few months because of the continuous reduction in the periapical lesion width. The lesion width reached a small value at 3 months and nearly reached zero at 6 months of follow-up, which indicated that all lesions had progressed and cured during the investigated examinations.

The results revealed that there were significant differences among the horizontal dimensions of periapical lesions on digital radiographs at the different investigated time points (*P* < .05).

The horizontal dimensions were successively reduced at each periodic radiographic measurement. This proves that the healing process has successfully and firmly progressed.

The differences in the width of the periapical lesions are displayed and analyzed in Table 2.

**Table 2:** Differences in the width of the periapical lesions on the radiographs obtained through periodic examinations

	Test Statistic	Standard Test Statistic	Significance	Adjusted Significance⁑
T_6_ -T_3_	0.500	0.775	0.439	1.000
T_6_ -T_2_	1.250	1.936	0.053	0.528
T_6_ -T_1_	2.583	4.002	< 0.001	0.001*
T_6_ -T_0_	3.583	5.551	< 0.001	0.000*
T_3_ -T_2_	0.750	1.162	0.245	1.000
T_3_ -T_1_	2.083	3.227	0.001	0.012*
T_3_ -T_0_	3.083	4.777	< 0.001	0.000*
T_2_ -T_1_	1.333	2.066	0.039	0.389
T_2_ -T_0_	2.333	3.615	< 0.001	0.003*
T_1_ -T_0_	1.000	1.549	0.121	1.000

T_0_, T_1_, T_2_, T_3_, and T_6_ Periapical lesion width at the beginning of treatment and at 1, 2, 3, and 6 months, respectively

⁑ Significance values were adjusted by the Bonferroni correction for multiple tests

* *P* < .05, Dunn-Bonferroni post hoc correction

The Dunn–Bonferroni post hoc correction revealed that there were significant differences among the five pairs of values at the examined periodic measurements (*P* < .05). This result proves that the curing process has been continuously occurring in a positive direction, with significant improvement occurring through every successive periodical examination.

The dimensional differences between the two successive periodic examinations are displayed in Table 3.

**Table 3:** The dimensional differences in the horizontal dimensions of the periodontal lesion on the digital radiographs between the two successive periodic examinations

	Minimum	25th percentile	Median	75th percentile	Maximum	Friedman test
T_0 − 1_	0.8000	1.382	2.095	3.897	6.480	< 0.001817*
T_1 − 2_	0.1100	0.677	1.285	2.457	4.090	
T_2 − 3_	-0.9500	0.000	0.865	2.482	3.480	
T_3 − 6_	0.0000	0.000	0.000	0.830	2.450	

T_0 − 1_, T_1 − 2_, T_2 − 3_, and T_3 − 6_ differences in lesion width between the two successive periodic examinations at the beginning and the first month,the first and the second month, the second month and the third month, and the third month and the sixth month, respectively

* P < .05, Friedman test

There were significant differences in the width of the periapical lesions among the periods of periodic examination (*P* < .05).

The differences in the width of the periapical lesion between the two successive periodic examinations are further analyzed in Table 4.

**Table 4:** Differences in the width of the periapical lesion on the radiographs obtained through periodic examinations

	Test Statistic	Standard Test Statistic	Significance	Adjusted Significance⁑
T_3 − 6_ - T_2 − 3_	0.583	1.107	0.268	1.000
T_3 − 6_ - T_1 − 2_	0.958	1.818	0.069	0.414
T_3 − 6_ - T_0 − 1_	1.958	3.716	0.000	0.001*
T_2 − 3_ - T_1 − 2_	0.375	0.712	0.477	1.000
T_2 − 3_ - T_0 − 1_	1.375	2.609	0.009	0.055
T_1 − 2_ - T_0 − 1_	1.000	1.897	0.058	0.347

T_0 − 1_, T_1 − 2_, T_2 − 3_, and T_3 − 6_ differences in lesion width between the two successive periodic examinations at the beginning and the first month, the first and the second month, the second month and the third month, and the third month and the sixth month, respectively

⁑ Significance values were adjusted by the Bonferroni correction for multiple tests

*P < .05, Dunn-Bonferroni post hoc correction

The Dunn–Bonferroni post hoc correction revealed that there was a significant difference in one pair of measurements between the two successive periodic examinations for each period of periodic examination. The results demonstrated that the lesion dimensions decreased rapidly in the first month and then decreased at a slower rate in the last month.

Between the 3- and 6-month periodic examinations, the mean difference in the reduction in lesion width was nearly zero, which indicated that there was no further room for reduction in lesion width. The differences in the decreases in the first period are greater than those in the last period, which means that the healing process is rapid in the first month.

The first signs of continuing formation of the root end were observed in the second month after completion of the procedure. The continuing creation of the root end in the present study has proven that this nonsurgical endodontic procedure is definitely the regenerative approach, according to contemporary knowledge. This firm evidence has been fundamental for modern knowledge in the evaluation of certain approaches to regenerative concepts in root canal therapy.

## Discussion

The results of the present study showed that all treated patients healed at different levels at periodical evaluations of both periapical digital radiographs and clinical signs.

Table 1 reveals that there are significant differences in lesion width among the different examination times according to the digital radiographs, and these measurements were continuously reduced throughout the investigated periods. The difference in lesion width occurred immediately after the first month, regardless of sex, age, or tooth type. The results of Table 2 reveals that the amplitude of this difference is greater than that of the other fluctuations during the following periodic examinations. These results prove the strong reaction of periapical lesions to the combination of the two modalities of PRF and MTA and indicate another proper treatment option for teeth with open apices and periapical radiolucent lesions. On the radiographs, not only was the horizontal dimension reduced but also the vertical dimension decreased. The periapical lesion image was changed from a radiolucent area to a radiopaque area throughout the examination period. Along with the change in the periapical digital radiograph, clinical symptoms did not occur immediately after the first month, and there were no other negative clinical signs on subsequent periodic examinations. Therefore, the success rate of nonsurgical endodontic procedures can reach 100%.

The procedure that was described in the present study is independent of root canal enlargement, which requires rotary nickel-titanium instruments [11, 12]. The irrigation protocol is important for this kind of nonsurgical endodontic procedure. All disinfected solutions were kept inside the root canal space to ensure no further irritation of the periapical tissue. This is a difficult task because of the wide open apical foramen facilitating the outflow of the irrigation solution at all times. These irrigation solutions should be sufficiently and properly activated to obtain enough water. The concentration of the irrigation solution should not have been high, as approximately 2% is reasonable.

A dental operating microscope with proper magnification is essential, indispensable, and invaluable in this delicate, complicated, and difficult procedure. Almost normal routine instruments could have been used for this special procedure, as long as the field of view was clear and illuminated enough. Unless the MTA carrier is used for insertion into the apical region, there is no further need for any other special instruments, as with microsurgical procedures under a dental operating microscope, where special microinstruments are indispensable [17]. Manipulation of a dental operating microscope for the present procedure has made working length determination less complicated [18]. The operator could clearly observe the length of the root canal from the occlusal surface through the apical foramen. The length of the root canal could be measured directly using a proper plugger and reconfirmed via periapical digital radiography. The electronic apex locator becomes redundant in these circumstances.

Illumination has been a very important factor in this nonsurgical endodontic procedure. Manipulation of the periapical region in the present protocol requires maximum light intensity to ensure clear vision in the field of view [17, 19]. However, periapical tissue should not be illuminated with high light intensity because this is a living tissue; much or less, it has been affected by high light intensity. Therefore, the field of view was intermittently illuminated by the on and off buttons or by the light intensity change knob on the body of the dental operating microscope. Sometimes, the right direction of the light into the periapical region is modified simply by changing the angle of the dental mirror whenever the operator desires it. The contrast filter of the dental operating microscope is sometimes used for temporarily adjusting the light intensity. Because almost all the teeth recruited into the study were lower teeth, the posture of the mandibular and, therefore, the position of the head of the patient was adjusted to the uncomfortable location. However, patient cooperation has been very good for nonsurgically sensitive endodontic treatment [17, 20].

The fluid squeezed from the PRF membranes was clearly observed by the operator and could be evaluated exactly until the PRF membranes were sufficient for the beginning of MTA placement.

Indirect vision in the dark, wet, complicated apical region requires a powerful light source and suitable magnification that cannot be achieved by conventional dental light or a normal head loupe even with enhanced illumination. This procedure is not difficult if the operator has been fully trained in a reasonable amount of time [17, 18, 20].

Blood sample preparation is a rather uncomfortable issue for adolescents and their parents in Vietnamese culture. A sample of ten millimeters of blood is a barrier to parental consensus because of the fear of disease. An increase in the amount of blood is uncomfortable for parents [21]. Several patients declined to enrol in the study because of the ten millimeters of blood sample needed for nonsurgical endodontic procedures. For fresh and immediate PRF blocks to manipulate the process, blood is withdrawn from the patient simultaneously with the endodontic progress to ensure a smooth journey from the beginning of the procedure to the insertion of pieces of PRF membranes into the periapical region [21].

Once the PRF block was extracted, it was then placed under the cover of the box for two minutes to create the PRF membrane. Preparation of the root canal and periapical region was accomplished before the PRF membrane was cut into 3 mm × 3 mm pieces for easier insertion into the periapical region.

The PRF membrane used in the present study was constructed with a box instrument accompanied by a centrifuge machine [22]. The steel plane is properly designated for the formation of the PRF membrane once the membrane is withdrawn from the vacuum sterile tube. The dimension of the piece of the PRF membrane correlates with the size of the apical foramen for each circumstance and facilitates the manipulation of the insertion through the foramen. The size of the plugger is suitable for the apical foramen because it does not further damage the delicate dentinal wall of the apical third [11, 12].

The patient posture during the procedure is certainly uncomfortable because of the time-consuming nature of the procedure and the unfavorable mandibular tooth position [19].

The results of the present study revealed that although PRF is an autogenous substance, the healing process time is not rapid enough compared with that of the collagen membrane used in a previous study [1]. Although PRF has certain advantages over collagen membranes, the manipulation of this autogenous material requires a strictly sterilized environment [11], leading to difficulty in normalizing the procedure in common clinical situations.

Once the apical barrier of the PRF membrane has been confirmed, the insertion of MTA into the desired position in the apical third of the root canal has not been further complicated. MTA could be carried out using an MTA carrier, a hand spiral instrument, or even ultrasonic energy, with the hope of a better result [23].

The MTA used for the apical plug was chosen for discoloration of the dental structure, especially for the anterior tooth. Although calcium silicate-based cement is placed only in the apical third, discoloration of the coronal portion of the tooth should be considered an adverse effect during this procedure [10]. Modern MTA erases the discoloration of teeth and is a reasonable reason for its use in the clinical setting. The moisture environment required for the setting of the conventional MTA was eliminated during the manipulation of this modern MTA; therefore, the following appointment for treatment completion was unnecessary. Water is rapidly separated from the PRF membrane; therefore, this substance should be manipulated as soon as possible to establish a parched region, facilitating subsequent MTA placement. The volume of the PRF barrier should be extended further into the apical region to compensate for the shrinkage of the PRF block after compression by sterile cotton.

The apical MTA barrier combined with the PRF membrane offers a high chance of successful restoration of extremely weakening tooth structure compared with other long-term, multiple visits, such as calcium hydroxide apexification and apical closure regeneration. Once the tooth has been fully restored on time, its functional, aesthetic, and stable abilities can be restored and enhanced in terms of both function and aesthetics, ensuring the best outcome for this kind of nonsurgical conservative endodontic procedure [24].

The texture of the PRF membrane seems tougher than that of the collagen membrane under the conditions of these PRF extraction parameters. This characteristic facilitates the manipulation of PRF pieces in the delicate apical region, although fluid separates from the compressed substance during placement [22].

The estimation of the blood volume needed for PRF extraction is not an issue if a previous cone-beam computed tomography image is available. The dimensions of the periapical lesion could be estimated more exactly via CBCT than via conventional periapical digital radiography, as described in the present study. CBCT is a very good method for evaluating the reduction of periapical lesions in all three dimensions in space. Further investigations using CBCT should be performed to obtain additional details on the healing process of this nonsurgical endodontic procedure. However, there is concern as to the radiation exposure used in CBCT capture to ensure the As Low As Reasonably Achieve (ALARA) principle [25].

PRF loaded with antibiotics after oral surgery has been advocated for in recent studies because of its many advantages [26].

The firm evidence of root apex development in the present study contributes to the contemporary knowledge of the regenerative approach in endodontics.

The limitations of the present study include the small sample size, short follow-up period, small number of teeth, and the use of only periapical digital radiographs. Further investigations should be performed using other calcium silicate-based materials, other PRF forms, and a broader range of teeth and ages. Fluid separation from the PRF membrane should be investigated to confirm that there is no considerable effect on the quality of the PRF membrane compared to that of the PRF block.

The PRF membrane is a good autogenous substance for the apical barrier formation procedure for preventing apical extrusion of material used in the apical region.

## Data Availability

No datasets were generated or analysed during the current study.

## Abbreviations

PRF: platelet-rich fibrin
RCF: relative centrifugal force
RPM: Rotations Per Minute
L-PRF: Leukocyte Platelet Rich Fibrin
A-PRF: Advanced platelet-rich fibrin
MTA: mineral trioxide aggregate
CBCT: Cone-beam computed tomography
ALARA: As Low As Reasonably Achieve
