# Correction: Platelet rich fibrin and MTA in the treatment of teeth with open apices

**DOI:** 10.1186/s12903-024-04087-y

**Published:** 2024-03-09

**Authors:** Van-Khoa Pham, Tran-Lan-Khue Pham, An-Tran Pham, Hoang-Lan-Anh Le, Thi-Bich-Van Tran, Manh-Cuong Hoang, Ta-Binh Vo, Khanh-Ngoc Vy, Minh-Hong Tran, Thi-Anh-Thu Tran, Minh-Anh Bui, Anh-Dung Hoang, Ngoc-Phuc Nguyen, Thi-Tam-Duyen Nguyen, Phuc-Nguyen Nguyen, Thi-Tuong-Vi Tran, Cao-Hoai-Linh Nguyen

**Affiliations:** 1 Faculty of Odonto-Stomatology, University of Medicine and Pharmacy at Ho Chi Minh City, Ho Chi Minh City, Vietnam; 2 National Hospital of Odonto-Stomatology, Ho Chi Minh City, Vietnam; 3 Faculty of Dentistry, Van Lang University, Ho Chi Minh City, Vietnam; 4 Faculty of Odonto-Stomatology, Tra Vinh University, Tra Vinh, Vietnam; 5 Hospital of Odonto-Stomatology, Ho Chi Minh City, Vietnam

**Correction to: BMC Oral Health (2024) 24:230**

10.1186/s12903-024-03923-5

The Funding information section was missing from this article [1] and should have read as below.
